# Inclusive palliative care for LGBTQIA+ individuals: A socioecological perspective on barriers and enablers

**DOI:** 10.1017/S1478951525100898

**Published:** 2025-10-13

**Authors:** Marta Almeida-Godinho, Paulo Reis-Pina

**Affiliations:** Faculty of Medicine, Center for Palliative Medicine, University of Lisbon, Lisbon, Portugal

**Keywords:** Diversity, equity, inclusion, health service needs and demands, LGBTQIA+ persons, palliative care, qualitative research

## Abstract

**Introduction:**

The LGBTQIA+ community faces pervasive discrimination, including in healthcare settings. This discrimination can be particularly detrimental during hospice and palliative care, where patients are especially vulnerable and may have distinct needs related to their sexual orientation or gender identity.

**Objectives:**

This study aimed to identify the barriers and enablers to accessing equitable and inclusive palliative care for LGBTQIA+ individuals.

**Methods:**

A self-administered online survey was conducted in November 2023 among LGBTQIA+ adults residing in Portugal. Thematic analysis was applied to identify barriers and enablers, mapped using an adapted socioecological framework.

**Results:**

Fifty-five respondents participated, primarily cisgender women (49.1%) identifying as homosexual (50.9%), with most aged 18–34 (76.4%). Barriers included caregiver homophobia, lack of LGBTQIA+-specific knowledge among professionals, fear among patients, misaligned care priorities, exclusion of partners from decision-making, and limited access to care. Enablers involved professional LGBTQIA+-specific training, psychological support, integration of partners or chosen families in care, workforce diversity, dissemination of palliative care information, community engagement, and inclusive societal values.

**Significance of results:**

Inclusive and responsive palliative care is essential to addressing the unique needs of LGBTQIA+ individuals. The findings highlight the need for systemic reforms to advance equity in care. The study calls for mandatory LGBTQIA+-focused training for healthcare providers, recognition of chosen families in care decisions, and public health campaigns that promote inclusivity. Collaboration with LGBTQIA+ organizations to improve outreach and access is vital, along with legislative measures to ensure equitable and inclusive care.

## Introduction

### Problem formulation

Palliative care is a specialized form of medical care aimed at improving the quality of life for patients with serious, often life-threatening illnesses, by alleviating physical, emotional, social, and spiritual suffering for both the patient and their family (World Health Organization 2020).

LGBTQIA+ refers to lesbian, gay, bisexual, transgender, queer or questioning, intersex, asexual, and more, encompassing a range of sexual orientations and gender identities (The Center 2024).

Despite ongoing efforts to combat prejudice, the LGBTQIA+ community remains marginalized and stigmatized, facing social stigma, discrimination, and systemic barriers (Clark 2014; Eschliman et al. 2024; Hatzenbuehler et al. 2024; Maltempi et al. 2025).

LGBTQIA+ patients often hesitate to disclose their sexual orientation or gender identity to healthcare providers, and may also encounter professionals who lack cultural competence in addressing their specific needs (Burton et al. 2020; Koch et al. 2023; Yu 2023). Additionally, LGBTQIA+ individuals often rely on friends or neighbors – commonly referred to as “chosen family” – for emotional and caregiving support when they experience rejection from biological families. Unfortunately, these chosen family members frequently lack legal recognition as next of kin, complicating decision-making and access to services in serious illness contexts (Jackson Levin et al. 2020; Knauer 2016; Stinchcombe et al. 2017).

Over time, medicine has evolved to be more person-centered, with an increasing focus on personalized care (Forsgren 2025). Nevertheless, significant gaps remain in addressing the vulnerabilities and specific needs of LGBTQIA+ patients within healthcare services, including palliative care.

### Purpose

The LGBTQIA+ community frequently encounters significant challenges in accessing appropriate palliative care services, which can adversely affect their health outcomes and even life expectancy. Given the limited research in this area, this study aimed to identify the potential barriers and enablers to palliative care for LGBTQIA+ individuals.

## Methods

This study was reported in accordance with the “Standards for Reporting Qualitative Research” (O’Brien et al. 2014). A completed checklist following these standards is included as Supplementary File 1.

### Qualitative approach and research paradigm

This study employed a qualitative, descriptive approach, collecting data through an online survey analyzed using inductive thematic analysis (Terry et al. 2017). This method was chosen to allow themes to emerge organically from the data, reflecting the exploratory nature of research on barriers and enablers to palliative care for LGBTQIA+ individuals.

We positioned this study within a constructivist/interpretivist paradigm to understand how LGBTQIA+ individuals construct meaning from their experiences with palliative care in Portugal’s specific cultural and social context. We applied the identified themes to Bronfenbrenner’s (1979) socioecological framework to explore the complex interactions among various contextual factors. This model conceptualizes healthcare access and decision-making across 5 levels: microsystem, mesosystem, exosystem, macrosystem, and chronosystem. For this study, these levels were adapted to the palliative care context, following Davidson et al. (2016): (1) individual, (2) interpersonal, (3) organizational, (4) community, and (5) societal/policy/system influences (Table 1). This framework facilitated a systematic analysis of barriers and enablers to PC for LGBTQIA+ individuals.

**Table 1.** Socioecological framework for barriers and enablers to palliative care as reported by LGBTQIA+ participants

Level	Barriers	Enablers
**Individual**	– Homophobia/prejudice	– Inclusivity
	– Lack of LGBTQIA+ knowledge	– Respect
	– Fear and vulnerability	
**Interpersonal**	– Partner involvement in decision-making	– Patient and partner involvement
**Organizational**	– Different treatment priorities	– Adequate training
	– Access to palliative care	– Workforce composition
		– Psychological support and trust-building
**Community**	– No barriers identified	– Increased information on palliative care
		– Community engagement
		– Moral values

### Researcher reflexivity

Our study was informed by existing literature, though perspectives from Portugal were limited. To address this, we adopted an inductive thematic analysis, allowing themes to emerge organically while remaining open to unexpected findings.

Data collection through an anonymous online survey minimized direct interaction, reducing potential researcher bias. Aware of our outsider status as physicians to some lived experiences shared by participants, we carefully addressed potential blind spots during analysis. This ensured participants’ voices, rather than our preconceptions, shaped the interpretation. While our professional background provided context, we prioritized participant-driven insights to minimize its influence on the study’s findings.

### Context and sampling strategy

Participants were recruited through various LGBTQIA+ organizations, including “ILGA Portugal,” “Dezanove,” and “Casa T Lisboa.” “ILGA Portugal” (*Intervention group for Lesbian, Gay, Bisexual, Trans, and Intersex People*), founded in 1995, is the country’s oldest and largest nongovernmental organization advocating for LGBTQIA+ rights and providing community support services. “Dezanove” is a Lisbon-based LGBTQIA+ media and advocacy platform that promotes visibility, cultural awareness, and social inclusion through journalism, campaigns, and community engagement. “Casa T Lisboa” is a community center that offers support, advocacy, and safe space initiatives for transgender and nonbinary individuals, with a focus on empowerment, health, and social integration.

These LGBTQIA+ advocacy organizations disseminated the survey link through their networks between 1 November 2023 and 30 November 2023.

*Inclusion criteria*: Participants were eligible if they: (1) were 18 years of age or older; (2) self-identified as gay, lesbian, transgender, gender diverse, or as having intersex variations; and (3) resided in Portugal.

Participants who identified as heterosexual, but also as transgender or gender fluid, were included in the study.

Individuals who completed less than 25% of the survey were excluded from the study.

### Ethical issues pertaining to human subjects

***Ethics approval***: This study was approved by the Ethics Committee of our Faculty of Medicine (no reference number, marked as “approved”). All ethical guidelines were strictly followed to protect participants’ rights and confidentiality. The study adhered to core ethical principles, including those outlined in the Declaration of Helsinki.

***Informed consent to participate***: Participation was voluntary, anonymous, and unpaid. Informed consent was implied upon survey initiation.

***Consent for publication***: Participants were informed, prior to starting the survey, that results would be used for scientific publication. By proceeding, they acknowledged and authorized this use, with the assurance of strict confidentiality and anonymity.

***Confidentiality***: LGBTQIA+ advocacy organizations distributed the survey link within their networks. Researchers had no access to participants’ personal data or email addresses.

### Data collection methods

Data were collected through a self-reported online survey using Google Forms® from November 1, 2023 to November 30, 2023. The survey was adapted from the study by Roberts et al. (2022) and included open-ended questions covering several areas: demographic characteristics, knowledge and opinions about palliative care, experiences with palliative care services, LGBTQIA+ identity within healthcare services, and the level of support provided by the National Health Service.

### Data processing

Both authors were actively involved in the data processing, which included data management, and security, as well as verification of data integrity, coding, and the anonymization/de-identification of excerpts. A Microsoft Excel® 16.0 spreadsheet was used to collate and organize the data, facilitating the categorization of themes.

### Data analysis

Both researchers conducted the data analysis using an inductive thematic approach. Initially, each researcher independently reviewed the survey responses to generate open codes that captured meaningful segments of text. Codes were iteratively refined, compared, and grouped into broader categories, which were then synthesized into overarching themes. The identified themes were subsequently mapped to an adapted socioecological framework for the palliative care context. This framework included 5 levels: (1) individual; (2) interpersonal; (3) organizational; (4) community; and (5) societal/policy/system level (Table 1). The authors discussed the coding, categorization, and theme development collaboratively, reaching consensus on their placement within the framework. Data management and coding were supported by MAXQDA 2022 (VERBI Software, Berlin, Germany) which facilitated the systematic organization and retrieval of data.

### Techniques to enhance trustworthiness

To ensure trustworthiness and credibility, we employed multiple strategies. Both researchers thoroughly reviewed the analysis, verifying coding and theme categorization against participants’ responses to maintain accuracy and consistency. Regular discussions promoted inter-rater reliability, resolved discrepancies, and enhanced the depth of interpretation while mitigating potential biases.

Member checking was conducted in collaboration with community representatives. Two representatives from each LGBTQIA+ organization that supported recruitment (ILGA Portugal, Dezanove, and Casa T Lisboa) voluntarily reviewed the preliminary findings. Their role was to assess whether the interpretations accurately reflected the experiences of LGBTQIA+ individuals, thereby strengthening the credibility of the analysis. This process did not involve re-contacting study participants and thus preserved their anonymity. Member checking confirmed the validity of the themes; while no major revisions were required, minor refinements in wording and emphasis were made to improve clarity.

## Results

### Participant characteristics

Fifty-eight people responded to the survey, with three exclusions, leaving a total of 55 participants (Table 2). Most were aged 25–34 (47.3%) and 18–24 (29.1%), with only 2 participants aged 45 or older. The most common gender identity was cisgender women (49.1%), followed by cisgender men (32.7%). Overall, 50.9% identified as homosexual (12 gay men and 16 lesbians).

**Table 2.** Participant characteristics (*n* = 55)

Characteristics	*N*	%
**Age (years)**		
18–24	16	28.6
25–34	26	47.3
35–44	11	19.6
≥45	2	3.6
**Gender**		
Cisgender woman	27	49.1
Cisgender man	18	32.7
Nonbinary/gender fluid	8	14.5
Transgender man	2	3.6
**Sexual orientation**		
Homosexual	28	50.9
Bisexual	16	28.6
Pansexual	9	16.1
Heterosexual	2	3.6
**Residency**		
Urban area	51	91.1
Rural area	4	7.3
**Nationality (country of birth)**		
Portuguese	47	85.5
Brazilian	6	10.7
Swiss	2	3.6
**Profession and fields of study**		
Provision of services (informatics, hospitality, sales and marketing, language services, teaching, police force, and cabin crew)	12	21.8
Medicine	10	18.2
Art-related professions (actor, artist, dancer, audiovisual creator, designer)	8	14.5
Nursing	5	9.1
Social sciences (archaeology, architecture, geography, management, and administration)	5	9.1
Health – other professions	4	7.3
Psychology	4	7.3
Student	4	7.3
Legal practice	2	3.6
Unemployed	1	1.8

Most participants were Portuguese (85.5%) and lived in urban areas (92.7%). Nearly 42% were healthcare professionals, with others working in service provision (20%) and the arts or social sciences (23.6%).

### Knowledge and experience with palliative care

All participants had heard of palliative care, though 17 (30.9%) were unsure of its meaning. All healthcare professionals were familiar with it. Most participants (*n* = 48, 85.7%) had no direct experience with palliative care; 3 had attended palliative care services, 2 knew an LGBTQIA+ person who had received palliative care, and 1 reported that their partner had received palliative care in the past 10 years.

### Barriers identified by LGBTQIA+ persons

Six categories of barriers were identified: (1) homophobia and prejudice; (2) lack of LGBTQIA+ knowledge; (3) patient fear and vulnerability; (4) different priorities in treatment and care; (5) partner involvement in decision-making and care; and (6) access to palliative care.

The first 3 relate to individual concerns (homophobia and lack of knowledge from healthcare professionals, and patient vulnerability), the next 2 to the interpersonal level, and the final barrier to organizational issues. These barriers were reported by both those with and without experience with palliative care. Three participants found no barriers related to their sexual or gender identity. Details by sexual orientation, gender, and location are in Table 3, with additional quotes in Table 4.

**Table 3.** Barriers to palliative care for LGBTQIA+ people (*n* = 55)

			Homophobia/ Prejudice	Lack of LGBTQIA+ knowledge	Patient fear and vulnerability	Different priorities in treatment and care	Partner involvement in decision-making	Access to palliative care	None	Does not know
**Gender**	**Cisgender woman (*N* = 27)**	***N***	22	5	2	2	4	1	2	0
		**%**	81	19	7	7	15	4	7	0
	**Cisgender man (*N* = 18)**	***N***	13	4	2	0	2	1	1	1
		**%**	72	22	11	0	11	6	6	6
	**Nonbinary (*N* = 8)**	***N***	6	1	0	1	2	0	0	1
		**%**	75	13	0	13	25	0	0	13
	**Trans man (*N* = 2)**	***N***	1	1	0	0	0	0	0	0
		**%**	50	50	0	0	0	0	0	0
**Sexual** **orientation**	**Homosexual (*N* = 28)**	***N***	18	7	3	0	5	2	3	1
		**%**	64	25	11	0	18	7	11	4
	**Bisexual (*N* = 16)**	***N***	13	3	1	2	1	0	0	1
		**%**	81	19	6	13	6	0	0	6
	**Pansexual (*N* = 9)**	***N***	9	1	0	1	2	0	0	0
		**%**	100	11	0	11	22	0	0	0
	**Heterosexual (*N* = 2)**	***N***	2	0	0	0	0	0	0	0
		**%**	100	0	0	0	0	0	0	0
**Location**	**Urban (*N* = 51)**	***N***	39	11	4	2	7	2	3	1
		**%**	76	22	8	4	14	4	6	2
	**Rural (*N* = 4)**	***N***	3	0	0	1	1	0	0	1
		**%**	75	0	0	25	25	0	0	25
**Total (*N* = 55)**		***N***	42	11	4	3	8	2	3	2
		**%**	76	20	7	5	15	4	5	4

**Table 4.** Participant quotes of barriers to palliative care (*n* = 55)

Barriers	Gender, sexual orientation, location	Examples of participant quotes
Homophobia/Prejudice	Cisgender woman, bisexual, urban	“Heteronormativity and transphobia manifest when there is an assumption that all patients are heterosexual and an emphasis on their biological sex, leading to a refusal to approach them according to their gender identity.”
	Cisgender man, homosexual, urban	“The way caregivers communicate with patients – often using neutral pronouns – reflects a lack of understanding on the part of the caregiver. Many caregivers feel constrained and uneasy when interacting with transgender individuals, particularly in sensitive situations like bathing, where they may encounter a trans woman with a penis or a trans man with a vagina.”
	Cisgender man, homosexual, urban	“When the illness is related to HIV, hepatitis, or other sexually transmitted infections, there is often a judgment associated with it.”
Lack of LGBTQIA+ knowledge	Trans man, bisexual, urban	“Healthcare professionals may struggle with specific questions related to my anatomy. For instance, urinary catheterization for a neopenis requires specialized knowledge, which, based on my experience, is often lacking among healthcare providers who are not specialized in that area.”
Patient fear and vulnerability	Cisgender man, homosexual, urban	“I believe that the need to feel safe, understood, and respected poses significant challenges. Many individuals may not feel comfortable being themselves in environments that lack openness, especially in vulnerable settings.”
	Cisgender man, homosexual, urban	“LGBTQIA+ patients may experience heightened sensitivity due to previous emotional stress, exacerbated by having grown up in a discriminatory society.”
	Trans man, bisexual, urban	“These individuals possess unique characteristics that make them more vulnerable, isolated, and less able to establish trusting relationships with caregivers.”
Different priorities in treatment and care	Nonbinary/fluid, bisexual, rural	“Transgender individuals undergoing hormone therapy may face issues regarding the respect of their medication needs.”
Partner involvement in decision-making and care	Nonbinary/fluid, pansexual, rural	“Family members may encounter difficulties accessing care due to their sexual orientation.”
	Nonbinary/fluid, homosexual, urban	“It is important to recognize and utilize mechanisms that acknowledge the patient’s chosen family.”
Access to palliative care	Cisgender woman, homosexual, urban	“Everyone should have equal access to healthcare and the same opportunities for care.”

As shown in Table 3, the most frequently reported barrier was homophobia/prejudice (76%), followed by lack of LGBTQIA+ knowledge among healthcare professionals (20%). Other barriers, such as patient fear and vulnerability, different treatment priorities, or limited partner involvement in decision-making, were reported less often. Although subgroup sizes were small, some patterns are worth noting. Nonbinary participants more often emphasized partner involvement in decision-making (25%), bisexual participants highlighted different priorities in treatment (13%), and all pansexual participants reported homophobia/prejudice (100%). Location (urban vs. rural) did not appear to meaningfully influence responses. These differences should be interpreted with caution given the limited sample sizes, but they provide useful descriptive insights into how barriers may vary across groups.

#### Homophobia and prejudice

The primary concern for participants was encountering homophobic healthcare providers. Issues ranged from heteronormative assumptions and disrespecting gender identity to deadnaming transgender patients, even when their legal documents reflected their correct name. A lack of empathy and understanding was also noted. Additionally, participants feared negligence, mistreatment, and judgment, especially when diagnosed with sexually transmitted infections, and ultimately, being treated differently from heterosexual cisgender patients.

#### Lack of LGBTQIA+ knowledge

A common barrier was the lack of LGBTQIA+ knowledge among healthcare providers, including confusion between gender identities and sexual orientations. This led to inappropriate questions that made patients feel uncomfortable or as if they were “social experiments.” Inadequate training, particularly in caring for transgender individuals, left healthcare professionals unfamiliar with diverse bodies, resulting in constrained or uncomfortable interactions with patients.

#### Patient fear and vulnerability

At the individual level, fear and vulnerability were significant barriers. LGBTQIA+ participants expressed feeling less safe in vulnerable situations, such as when seeking medical care, due to their identity.

#### Different priorities in treatment and care

At the interpersonal level, participants noted differing priorities between themselves and healthcare providers, particularly concerning transgender individuals and potential conflicts between hormone therapy and acute treatments. There was also concern about the lack of discussion around their desires and priorities, and the fear of being excluded from their own care decisions.

#### Partner involvement in decision-making and care

Participants were concerned that healthcare providers failed to recognize their partners or chosen family, leading to exclusion from decision-making. This also raised fears about being denied visitation during “family hours.”

#### Access to palliative care

At the organizational level, participants reported difficulties in accessing palliative care services, including limited availability of specialized providers, lack of clear referral pathways, and insufficient information about existing services. Some also described encountering delays or uncertainty about eligibility, which created additional barriers to timely care. At the same time, participants highlighted potential opportunities for improvement, such as expanding outreach efforts, strengthening referral systems, and ensuring that palliative care services are explicitly inclusive of LGBTQIA+ individuals.

### Enablers identified by LGBTQIA+ persons

Eight categories of enablers were identified: (1) inclusivity and respect; (2) patient and partner involvement; (3) psychological support and trust-building; (4) adequate training; (5) workforce composition; (6) increased information on palliative care; (7) community engagement; and (8) moral values. Of these, Enabler 1 is situated at the individual level, Enabler 2 at the interpersonal level, Enablers 3–5 at the organizational level, and Enablers 6–8 at the community level. These enablers were reported by participants with and without experience in palliative care. Details by sexual orientation, gender, and location are presented in Table 5, with additional quotes in Table 6.

**Table 5.** Enablers to palliative care for LGBTQIA+ people (*n* = 55)

			Inclusivity and respect	Patient and partner involvement	Psychological support	Adequate training	Workforce composition	Increased information on palliative care	LGBTQIA+ community engagement	Moral values	Does not know
**Gender**	**Cisgender woman (*N* = 27)**	***N***	16	2	2	7	0	20	2	1	3
		**%**	59	7	7	26	0	74	7	4	11
	**Cisgender man (*N* = 18)**	***N***	9	1	1	7	1	12	3	0	1
		**%**	50	6	6	39	6	67	17	0	6
	**Trans man (*N* = 2)**	***N***	2	0	0	0	0	2	0	0	0
		**%**	100	0	0	0	0	100	0	0	0
	**Nonbinary (*N* = 8)**	***N***	2	0	1	6	1	5	1	0	0
		**%**	25	0	13	75	13	63	13	0	0
**Sexual orientation**	**Homosexual (*N* = 28)**	***N***	15	2	2	10	1	17	5	0	2
		**%**	54	7	7	36	4	61	18	0	7
	**Bisexual (*N* = 16)**	***N***	8	1	1	7	0	14	0	0	1
		**%**	50	6	6	44	0	88	0	0	6
	**Pansexual (*N* = 9)**	***N***	5	0	1	2	1	6	1	1	1
		**%**	56	0	11	22	11	67	11	11	11
	**Heterosexual (*N* = 2)**	***N***	1	0	0	1	0	2	0	0	0
		**%**	50	0	0	50	0	100	0	0	0
**Location**	**Urban (*N* = 51)**	***N***	28	3	4	17	2	36	6	1	4
		**%**	55	6	8	33	4	71	12	2	8
	**Rural (*N* = 4)**	***N***	1	0	0	3	0	3	0	0	0
		**%**	25	0	0	75	0	75	0	0	0
**Total**		***N***	29	3	4	20	2	39	6	1	4
		**%**	53	5	7	36	4	71	11	2	7

**Table 6.** Participant quotes of enablers for palliative care (*n* = 55)

Enablers	Gender, sexual orientation, location	Examples of participant quotes
**Inclusivity and respect**	Cisgender woman, pansexual, urban	“The use of correct pronouns and the absence of judgment or taboo regarding the patient’s sexual choices.”
	Cisgender woman, bisexual, urban	“The use of neutral terms and avoiding a phallocentric approach to discussions about virginity, ensuring integration of lesbian and/or bisexual women while being respectful of individuals’ bodies and identities.”
	Cisgender woman, homosexual, urban	“Equality of rights and treatment is essential. I feel there is still significant unfamiliarity regarding these issues.”
	Cisgender woman, homosexual, urban	“Using inclusive language that does not assume gender, identity, or sexual orientation based solely on a person’s gender expression.”
**Patient and partner involvement**	Cisgender man, homosexual, urban	“Identifying the needs of individuals and involving the community in service provision and healthcare decision-making.”
	Cisgender woman, bisexual, urban	“Acting without discrimination and integrating the family unit into conversations and decision-making processes.”
**Psychological support**	Cisgender woman, bisexual, urban	“Providing adequate support tailored to the patient’s needs, maintaining openness throughout the process, and offering psychological support.”
	Cisgender man, homosexual, urban	“Creating preventive medicine centers for LGBTQIA+ patients, staffed by professionals trained to address their specific needs. Support centers for victims of medical violence would also be beneficial.”
**Adequate training**	Cisgender woman, bisexual, urban	“Having a medical team that is familiar with LGBTQIA+ individuals and their needs would be a positive step, alongside basic requirements such as empathy.”
	Cisgender man, homosexual, urban	“Equipping healthcare personnel with comprehensive information to prevent situations that could cause discomfort, shame, or insult to any patient, particularly those who are LGBTQIA+.”
	Nonbinary/fluid, bisexual, rural	“Educating health professionals and everyone involved in the support network about the LGBTQIA+ community.”
	Cisgender woman, bisexual, urban	“Providing training for healthcare professionals to help them understand the realities faced by their patients.”
	Nonbinary/fluid, bisexual, urban	“Training staff on LGBTQIA+ issues, especially related to transgender topics, to ensure they can approach patients without causing discomfort.”
**Workforce composition**	Cisgender man, homosexual, urban	“A service staffed by peers, where the individuals attending to me are also part of the LGBTQIA+ community.”
	Nonbinary/fluid, pansexual, urban	“Inclusivity requires training for palliative care providers, but true inclusivity involves having service deliverers who are also part of the LGBTQIA+ community. A discriminatory environment drives these individuals away from such professions, which needs to change to achieve genuinely inclusive services.”
**Increased information on palliative care services**	Cisgender man, homosexual, urban	“Family doctors are best positioned for direct patient contact and to promote information specifically for LGBTQIA+ patients.”
	Cisgender woman, pansexual, urban	“Promoting palliative care to the general population – not just the LGBTQIA+ community – through media campaigns that include LGBTQIA+ individuals, demonstrating that palliative care is equally available to everyone.”
**LGBTQIA+ community engagement**	Cisgender woman, homosexual, urban	“Aligning with spokespeople from the LGBTQIA+ community to convey a strong, unified message.”
**Community values**	Cisgender woman, pansexual, urban	“I don’t think the issue lies solely with palliative care; rather, it depends on society’s overall perception of the LGBTQIA+ community. Once discrimination is eliminated, fear of discrimination in healthcare will diminish.”

As shown in Table 5, the most commonly reported enablers were increased information on palliative care (71%), inclusivity and respect (53%), and adequate training (36%). Patterns differed slightly across subgroups, although interpretation is limited by small numbers. Non-binary participants more often emphasized adequate training (75%), while bisexual respondents highlighted increased information (88%). Pansexual participants identified inclusivity and respect (56%) and, unlike other groups, also mentioned moral values (11%). No major differences were observed between urban and rural participants, though rural respondents (*n* = 4) more frequently selected adequate training (75%). These findings should be interpreted cautiously but suggest that access to clear information, inclusivity, and provider training are consistently perceived as critical enablers.

#### Inclusivity and respect

The primary strategy for LGBTQIA+ patients to feel safe in health and palliative care is for providers to approach them with dignity and empathy. Healthcare providers should use neutral terms, correct pronouns, and names, avoiding assumptions about gender identity or sexual orientation. Overall, participants emphasized the importance of inclusivity.

#### Patient and partner involvement

Participants expressed a desire for greater involvement in their care and decision-making, advocating for open communication between patients and healthcare providers. They emphasized the need for romantic partners and chosen family to be included in decision-making, whether through simplifying bureaucratic processes for recognition at an organizational level or ensuring interpersonal acknowledgment.

#### Psychological support and trust-building

At the organizational level, psychological support must extend beyond individual counseling to include systemic efforts that foster trust and safety for LGBTQIA+ patients. Suggestions included establishing safe spaces within healthcare systems, creating medical centers specifically for LGBTQIA+ individuals, and developing support centers for victims of medical violence or neglect. Such initiatives contribute to rebuilding confidence in healthcare institutions while addressing the psychological impact of discrimination.

#### Adequate training

Healthcare providers require enhanced education on LGBTQIA+ issues, with specialized training designed to build both knowledge and competence in working with diverse identities and bodies. Such training should not only improve literacy and familiarity but also equip providers with the practical skills necessary to deliver affirming, inclusive, and culturally competent care.

#### Workforce composition

Ensuring equal employment opportunities for LGBTQIA+ providers is crucial, as their representation within the workforce can help patients feel safer and more understood.

#### More information on palliative care

At the community level, increased dissemination of information about palliative care is essential. This could include promoting available services through flyers, informational materials, and publicity on national health service websites and apps. Materials should incorporate inclusive photos and symbols – such as the rainbow flag – or other indicators of LGBTQIA+ affirming care. Greater investment in targeted, inclusive communication strategies is needed, alongside public campaigns designed to raise awareness and foster trust in palliative care among LGBTQIA+ communities.

#### LGBTQIA+ community engagement

Palliative care and other healthcare professionals should collaborate with community spokespeople to effectively amplify the voices and needs of LGBTQIA+ individuals. By positioning themselves as community allies, healthcare professionals can foster a welcoming environment, encouraging LGBTQIA+ individuals to access these services without fear or hesitation.

#### Moral values

Inclusivity in palliative care extends beyond the healthcare system and reflects broader societal attitudes. Eliminating homophobia, transphobia, and discrimination from cultural and social norms is essential, as these values inevitably shape healthcare interactions. However, this enabler is also the most difficult to achieve, as shifting societal attitudes often requires decades of sustained effort. Moreover, in many regions – including Portugal, the United States of America, and other parts of the world – recent legislation and public discourse demonstrate that discrimination against LGBTQIA+ people can persist or even increase. These realities highlight the importance of pairing structural and organizational interventions with long-term cultural change to ensure that palliative care environments become consistently inclusive and affirming.

### Synthesis of the socioecological framework adapted for the palliative care context

The potential barriers and enablers to accessible and appropriate palliative care for LGBTQIA+ individuals, mapped to the levels of the socioecological framework, are illustrated in Table 1.

## Discussion

### General interpretation of barriers in palliative care for LGBTQIA+ people

Although the subgroup analyses are limited by small cell sizes, some descriptive differences merit attention. Nonbinary participants emphasized partner involvement in decision-making more often than other groups, which echoes prior research highlighting the heightened vulnerability of nonbinary individuals to exclusion within healthcare decision-making (Bauer et al. 2019). Bisexual participants more frequently reported differing treatment priorities, aligning with studies suggesting that bisexual people often face unique forms of invisibility and erasure in clinical contexts (Ross et al. 2018). Finally, the finding that all pansexual respondents reported homophobia/prejudice is consistent with evidence that individuals with less widely understood sexual orientations may encounter amplified discrimination (Callis 2014). While these patterns cannot be generalized, they underscore the importance of considering the heterogeneity of experiences within LGBTQIA+ communities when designing inclusive palliative care interventions.

### General interpretation of enablers of palliative care for LGBTQIA+ people

Although subgroup analyses are limited by small sample sizes, several descriptive trends are noteworthy. Across the sample, enablers such as increased information about palliative care, inclusivity and respect, and adequate training were most frequently reported, reflecting broader findings that affirm that health literacy and culturally competent care are critical facilitators for sexual and gender minority groups in palliative settings (de Jong et al. 2024; Haviland et al. 2020). Notably, nonbinary individuals particularly emphasized the need for adequate training, resonating with research that highlights clinicians’ limited preparedness to serve nonbinary patients (Holland et al. 2024; Scandurra et al. 2019). Bisexual respondents’ prioritization of increased information mirrors literature suggesting bisexual people frequently face invisibility in healthcare environments and rely on clear, inclusive guidance (Ross et al. 2018). These descriptive insights, while preliminary, underscore the importance of affirming communication and provider competency as levers for inclusive palliative care.

### Individual level barriers and enablers

Participants expressed concerns about potential discrimination in healthcare and palliative care due to homophobic beliefs held by professionals. This discrimination can manifest as incorrect assumptions about sexual orientation or disregard for gender identity, including the use of incorrect names or pronouns. Participants feared negligence, mistreatment, and judgment from professionals simply for identifying as LGBTQIA+.

Our findings resonate with prior work documenting barriers faced by LGBTQIA+ individuals in serious illness and palliative care. Stein et al. (2020) reported that hospice and palliative care teams often encounter challenges in providing inclusive care for LGBTQ + patients and families, including inadequate training, heteronormative assumptions, and limited recognition of partners in decision-making. Similarly, Stein et al. (2023) found that seriously ill LGBTQ + patients and their partners described experiences of marginalization and exclusion by healthcare providers, highlighting the persistence of discrimination and lack of understanding in clinical encounters.

A widespread lack of knowledge regarding LGBTQIA+ issues, particularly related to transgender individuals and their unique anatomies, was highlighted. A scoping review noted significant knowledge gaps about transgender older adults (Stinchcombe et al. 2017). The reluctance to explore patients’ sexual orientation and gender identity hampers effective palliative care delivery (Hunt et al. 2019). Additionally, fear and vulnerability were prominent concerns in healthcare settings (Roberts et al. 2022). A systematic review identified fear of poor treatment as a major barrier to disclosing sexual orientation (Brooks et al. 2018). A Portuguese study found that 31% of LGBTQIA+ individuals had concealed their sexual orientation during medical appointments (Pinto et al. 2015). While anyone can feel vulnerable when ill, LGBTQIA+ patients often face added anxiety regarding anticipated discrimination, compounding their vulnerability in palliative care (Bristowe et al. 2018; Wakefield et al. 2021). This fear can deter gay men from seeking treatment or information about safe sex (Clark 2014). In palliative care, where patient comfort is paramount, such fears significantly hinder trust in professionals during critical moments.

To foster understanding and empathy, individual providers should actively enhance their knowledge of LGBTQIA+ issues, using neutral terms and the correct names and pronouns to promote inclusivity and respect. This is crucial, especially since only a minority of Portuguese healthcare providers ask sexuality and relationship questions that acknowledge LGBTQIA+ identities (Pinto et al. 2015). An example of inclusive language can be seen in a clinical case of a transgender man diagnosed with metastatic ovarian cancer, where professionals used terms like “germ cell tumor” and male reference values in line with his testosterone therapy (Stevens and Abrahm 2019).

Stein et al. (2020) noted that many providers expressed a strong commitment to delivering compassionate care and recognized the importance of inclusivity in practice. In *Project Respect*, Stein et al. (2023) demonstrated that respectful communication, acknowledgment of partners, and affirming provider behaviors were viewed by LGBTQ + patients as key enablers of trust and engagement with healthcare services. These findings are consistent with our results, underscoring that provider training, inclusivity, and respectful organizational practices are essential strategies for building LGBTQIA+-affirming palliative care environments.

Displaying LGBTQIA+-friendly symbols, such as pride flags, in waiting rooms or offices can signal a welcoming environment, reducing fears of discrimination (Pinto et al. 2015; Rosa et al. 2023). However, it is essential to ensure these gestures are genuine, as perceived inauthentic attempts at inclusivity can deepen distrust in healthcare systems among LGBTQIA+ individuals (Lintott et al. 2022).

### Interpersonal level barriers and enablers

Participants expressed concerns about differing priorities in health management between themselves and healthcare providers, leading to a lack of discussion about their preferences. Pang et al. (2019) similarly found that patients transitioning later in life prioritized transitioning-related concerns over end-of-life issues. When doctors overlook the significance of gender identity and sexuality to patients, it can foster mistrust and disengagement.

Participants also highlighted the inadequate recognition and involvement of their romantic partners, aligning with findings in Portuguese research (Pinto et al. 2015). Participants felt that LGBTQIA+ partners were often less involved in decision-making than heterosexual partners and raised concerns about visitation rights in hospital settings, despite legal provisions allowing any individual to visit during designated hours.

Given the prevalence of homophobia within some biological families, many LGBTQIA+ individuals cultivate chosen families, nonbiological kinship networks that provide vital support. However, these relationships are often not legally recognized, and participants feared that both romantic partners and chosen families would be excluded from communication about clinical information and decision-making.

Since June 5, 2010, Portugal has legally recognized same-sex marriage, granting full marital rights – including inheritance, hospital visitation, and decision-making authority – equal to those of opposite-sex spouses. Same-sex partners, therefore, possess the legal authority to participate in treatment decisions, access patient information, and assume guardianship roles when necessary. This legal framework represents an important structural enabler for the recognition of partners in healthcare and palliative care contexts.

Yet, despite these robust protections, challenges and barriers persist, as reflected in participants’ accounts. Cultural and social norms – rooted in heteronormative assumptions – continue to influence how LGBTQIA+ relationships are perceived by families, some healthcare professionals, and even institutional practices. In some cases, partners still encounter skepticism or inadequate acknowledgment of their role, particularly among older generations or in care settings lacking inclusive protocols. Thus, while the legalization of same-sex marriage has established a strong legal foundation, its potential is not fully realized without deliberate efforts: provider education, inclusive institutional policies, and broader cultural change are required to ensure that LGBTQIA+ partners and chosen families are consistently recognized as legitimate advocates in decision-making.

### Organizational level barriers and enablers

Access to healthcare can be a significant barrier for LGBTQIA+ patients in Portugal. Despite the national health system being public and accessible to all, past traumatic experiences and fear of discrimination may deter LGBTQIA+ individuals from utilizing services. According to “Associação ILGA Portugal,” nearly one-third of LGBTQIA+ participants hesitated to use national healthcare services, and 36% sought health information through non-in-person channels, such as the internet (Pinto et al. 2015). It is crucial to distinguish between access to healthcare and health equity; minority groups like the LGBTQIA+ community face additional barriers that require targeted support to achieve equitable health outcomes (Wakefield et al. 2021).

Participants proposed several organizational strategies to improve LGBTQIA+ access to palliative care, including enhanced psychological support. However, given the community’s vulnerability to discrimination, this support should be provided by professionals trained in LGBTQIA+ issues (Nunes 2019).

Participants also suggested creating dedicated health centers for LGBTQIA+ patients, where staff are trained to competently address diverse identities and bodies. Experiences such as misgendering, denial of care, coercive interventions, or disrespectful treatment practices can lead LGBTQIA+ individuals to perceive healthcare organizations as discriminatory, pathologizing, or unsafe (Poteat et al. 2013; Reisner et al. 2015). Establishing support centers focused on addressing traumatic events, minority stress, and microaggressions – including both interpersonal and institutional discrimination perpetrated by healthcare providers or peers in therapeutic contexts – could help rebuild trust in healthcare services (Livingston et al. 2019). This is particularly important because LGBTQIA+ individuals may also experience vicarious trauma through shared community narratives, which heightens their sense of vulnerability (Burton et al. 2020).

Further recommendations included professional training on LGBTQIA+ matters for healthcare providers. Resources should be allocated to integrate this training into the education of all health professionals and to provide workshops in hospitals for all staff who interact with patients. The objective is to create an environment where patients feel safe and respected regarding their identities.

Lastly, workforce diversity is essential. Employing LGBTQIA+ healthcare professionals can bridge gaps between patients and healthcare providers, fostering understanding of patient vulnerabilities and needs. Additionally, a more inclusive workforce may reduce discrimination and encourage other qualified individuals from the community to pursue careers in healthcare. A diverse workforce also provides visible role models and potential mentors for all staff, thereby strengthening the delivery of culturally competent and affirming care for LGBTQIA+ people.

### Community-level enablers

While no barriers were identified at the community level, participants highlighted several potential enablers. The first is increased dissemination of information about palliative care to both the general population and the LGBTQIA+ community. This can be achieved through websites, flyers, apps, and social media. Additionally, organizing workshops, public talks, and campaigns could clarify what palliative care entails, allowing people to address any doubts about end-of-life care and fostering trust, particularly among LGBTQIA+ individuals. Healthcare professionals could also play a vital role in disseminating information during appointments.

These strategies align with the recommendations of Acquaviva (2023), who underscores that building LGBTQIA-inclusive palliative care requires not only accurate information-sharing but also sustained engagement with community organizations. Collaborating with LGBTQIA+ groups in the design and delivery of outreach initiatives helps ensure that materials are culturally sensitive, affirming, and responsive to community needs. Such partnerships can enhance visibility, empower patients and families, and promote trust in healthcare systems. By embedding community perspectives into outreach and education, healthcare organizations can move beyond generic awareness campaigns toward practices that actively affirm LGBTQIA+ identities and lived experiences.

Participants suggested enhancing collaboration with community spokespeople and organizations to facilitate two-way information exchange regarding palliative care. Increased outreach efforts could help alleviate patient apprehension and demonstrate a commitment to inclusive care (Rosa et al. 2023).

Finally, shifts in moral values within the community toward greater tolerance and inclusivity could lead to more LGBTQIA+-friendly healthcare services. Although society has become more accepting over the years, LGBTQIA+ individuals still face marginalization. Moreover, in some countries, recent years have also seen increased negative attitudes and discriminatory practices toward LGBTQIA+ people – particularly against transgender individuals – demonstrating that progress is uneven and fragile. Therefore, additional strategies are necessary to ensure that LGBTQIA+ people feel safe and supported when accessing healthcare and palliative care services.

### Policy implications

Portugal offers a robust legal foundation for LGBTQIA+ rights (ECRI - The European Commission against Racism and Intolerance 2025). The Constitution of the Portuguese Republic, in its seventh revision, in 2005, explicitly prohibits discrimination based on sexual orientation. Law no. 38/2018 established the right to self-determination of gender identity and gender expression, and the right to the protection of a person’s sex characteristics. Law no. 85/2021 prohibited discrimination based on gender identity or sexual orientation in eligibility to donate blood. Law no. 15/2024 prohibited the so-called “conversion therapies.” In addition, the National Strategy for Equality and Non-Discrimination 2018–2030 includes specific measures to protect LGBTQIA+ individuals in healthcare (CIG - Comissão para a Cidadania e a Igualdade de Género 2018).

Portugal also legalized advance directives under Law No. 25/2012, enabling individuals to register future medical care preferences and appoint healthcare proxies. While this framework represents a significant enabler for LGBTQIA+ individuals, knowledge and uptake remain limited, underscoring the importance of targeted educational initiatives to ensure equitable access and use (Martins and Nunes 2023).

This research underscores the urgent need for policies to promote inclusivity in palliative care for LGBTQIA+ individuals. Key recommendations include:

- ***Healthcare training***: Mandatory LGBTQIA+-focused training modules for healthcare professionals to address knowledge gaps, particularly concerning gender identity and nontraditional family dynamics.
- ***Organizational guidelines***: Development of standards for LGBTQIA+ inclusivity within palliative care services, including the display of welcoming symbols and use of inclusive language.
- ***Outreach and awareness***: Collaboration with LGBTQIA+ organizations to provide accessible information about palliative care through workshops, campaigns, and primary care networks.
- ***Legislative measures***: Recognition of chosen families in healthcare decision-making processes, ensuring they have the same rights as biological families in end-of-life care.

Implementing these measures can reduce barriers, foster trust, and improve palliative care experiences for LGBTQIA+ individuals, ensuring equitable access and dignity at the end of life.

### Strengths and limitations

This study makes an original contribution by addressing a critical gap in the understanding of barriers and enablers to palliative care for LGBTQIA+ individuals in Portugal. Collaboration with LGBTQIA+ advocacy organizations enhanced both the relevance and credibility of the study, while also facilitating recruitment and ensuring that the community’s specific needs and concerns were represented. The use of an anonymous, voluntary online survey encouraged honest participation and helped reduce social desirability bias.

Ethical standards were rigorously observed, safeguarding participants’ rights, privacy, and confidentiality. Data were analyzed thematically, with findings cross-checked between researchers to ensure consistency and reliability in interpretation. Feedback was incorporated to validate the themes, further enhancing the authenticity of the conclusions. Importantly, the findings generate practical recommendations for healthcare providers, policymakers, and community stakeholders, thereby strengthening the applicability and potential impact of the study in advancing equity and inclusivity in palliative care.

This study presents several limitations that must be acknowledged. Initially, disseminating the survey proved challenging; however, support from LGBTQIA+ advocacy organizations significantly facilitated participant recruitment. Given the sensitive nature of the topic, overall participation was limited, resulting in a small sample size that may not adequately represent the diverse experiences of LGBTQIA+ individuals. Most participants were young and relatively healthy, with only 2 participants over the age of 45. In addition, a majority of participants were healthcare providers rather than patients or caregivers. This demographic profile may have skewed perspectives on palliative care, terminality, and end-of-life issues toward those of professionals or younger community members, rather than reflecting the lived experiences of older adults or individuals facing advanced, serious illness.

As a result, the voices of those most likely to need and use hospice and palliative care services – older LGBTQIA+ adults and patients with advanced illness – are largely missing from this dataset. These factors represent important limitations to the generalizability of our findings. Accordingly, the results should be interpreted with caution, and future research is urgently needed to capture the perspectives of older LGBTQIA+ populations and those directly navigating serious illness.

### Future research

Future research should build on these findings by conducting larger, mixed-methods studies to deepen understanding of the barriers and enablers to palliative care for LGBTQIA+ individuals. Comparative studies across different cultural and healthcare contexts would help identify both universal and context-specific needs. Longitudinal research could explore how experiences and outcomes evolve over time, while intervention studies are needed to test the effectiveness of LGBTQIA+-focused training programs, inclusive policies, and community–healthcare partnerships. Engaging LGBTQIA+ individuals and organizations in participatory research will be essential to ensure that future interventions remain responsive, equitable, and sustainable.

## Conclusions

This study identified key barriers and enablers to palliative care for LGBTQIA+ individuals in Portugal, using a socioecological framework. Barriers included discrimination by professionals, lack of LGBTQIA+ knowledge, and limited involvement of partners and chosen families, all of which contribute to fear and vulnerability.

At the same time, important enablers emerged, such as inclusive training for healthcare providers, supportive organizational policies, and stronger community engagement. These strategies can foster trust and create more affirming care environments.

Although limited by a small and unrepresentative sample, the study highlights the urgent need for targeted interventions to ensure equitable access and culturally competent palliative care. Addressing barriers and strengthening enablers is essential to improving the quality of care and affirming the identities and experiences of LGBTQIA+ patients at the end of life.

## Supporting information

10.1017/S1478951525100898.sm001 Almeida-Godinho and Reis-Pina supplementary material Almeida-Godinho and Reis-Pina supplementary material

## Data Availability

All data and materials are available within the article. No additional data is available beyond what is included in this publication.

## Supplementary material

The supplementary material for this article can be found at https://doi.org/10.1017/S1478951525100898.
